# Efficacy and safety of CYP2C19 genotype in stroke or transient ischemic attack patients treated with clopidogrel monotherapy or clopidogrel plus aspirin: Protocol for a systemic review and meta-analysis

**DOI:** 10.1097/MD.0000000000011060

**Published:** 2018-06-15

**Authors:** Jia-Chen Yao, Min Cui, Mang-Mang Pan, Zhi-Chun Gu, Wen-Yan Li

**Affiliations:** a Department of Pharmacy, Shanghai Pudong New Area Gongli Hospital; b Department of Pharmacy, Renji Hospital, School of Medicine, Shanghai Jiaotong University, Shanghai, China.

**Keywords:** clopidogrel, CYP2C19, meta-analysis, stroke

## Abstract

**Background:**

The relationship of CYP2C19 genotype and clinical efficacy in stroke or transient ischemic attack (TIA) patients treated with clopidogrel monotherapy or clopidogrel plus aspirin remains unknown. We thus aim to conduct a meta-analysis to appraise evidence on the association of CYP2C19 genotype and clinical efficacy for stroke or TIA.

**Methods:**

An electronic search will be performed for clinical trials that reported the interested efficacy data (stroke, myocardial infarction, or vascular death) and safety data (any bleeding) in clopigogrel-treated patients with stroke or TIA. Odds ratios (ORs) with their confidence intervals (CIs) will be calculated using a meta-analysis.

**Results:**

This study will provide the evidence of the relationship between CYP2C19 genotype and clinical efficacy and safety in stroke/TIA patients taking clopidogrel by pooling the results of individual studies.

**Conclusions:**

The results will bring about vigorous evidence in this issue and guide both clinical decision-making and future research.

## 1 Introduction

Stroke, as a complex neurological disease, kill approximately 5.7 million people each year according to the World Health Organization (WHO).^[1]^ About 80% of strokes are ischemic caused by occlusion of the intracerebral artery, and antiplatelet therapy taking clopidogrel alone or in combination with aspirin is commonly used for the secondary stroke prevention in at-risk individuals.^[2,3]^ However, there is a large degree of interindividual variability in pharmacodynamic response to clopidogrel.^[4]^ One source of the variability is the metabolism of clopidogrel, which is an inactive pro-drug that requires a 2-step biotransformation by a number of CYP450 isoenzymes to generate the active thiol metabolite.^[5]^ Mutations in the genes of the CYP450 enzymes may affect clopidogrel responsiveness, and CYP2C19 is of great concern.^[5]^ Accordingly, reduced-function CYP2C19 genotype has been associated with decreased antiplatelet effects and increased risk of major adverse cardiovascular events (MACEs) in both acute coronary syndromes and post percutaneous coronary intervention patients, which have been summarized in several systematic reviews abstracting data from genetic association studies.^[6–9]^ The reported summary effect estimates have confirmed that carriers of reduced-function allele had approximately 1.5 to 2 times the risk of occurrence of a MACE or stent thrombosis in patients with post-PCI compared with noncarriers.^[6–9]^ Yet, there is no consensus as to whether the diminished pharmacologic response leads to increased clinical adverse events in patients with ischemic stroke (IS) or transient ischemic attack (TIA). To date, very limited data are available addressing the effect of CYP2C19 gene variants on clopidogrel efficacy in IS or TIA.^[10,11]^ Therefore, to define efficacy and safety in carriers of reduced-function CYP2C19 allele, we aim to perform a systematic review and meta-analysis of the accumulated information of association studies between reduced-function CYP2C19 gene variants and clinical outcomes in patients with stroke or TIA.

## 2 Methods

### 2.1 Data sources and searches

We will adhere to the PRISMA statements for reporting systematic reviews and the guidance from the Human Genome Epidemiology Network for reporting gene-disease association studies.^[12,13]^ A comprehensive literature search (Medline, Embase, Cochrane Library electronic databases, Wanfang database, CKNI database, and VIP database) will be conducted to identify all potential eligible trials without language restrictions. The search will comprise the following terms: patients (Stroke or IS or transient ischemic attack or TIA); drug name (clopidogrel or Iscover or Plavix); and gene (gene or gene polymorphism or gene variant). References of all pertinent articles will also be scrutinized to ensure that all relevant studies are identified.

### 2.2 Study selection and eligibility criteria

The following inclusion criteria for study selection will be used: case–control studies, cohort studies, or clinical trials; IS or TIA patients treated with clopidogrel; studies including only adults aged > 18 years; the size of sample>50; and studies reporting relevant adverse events for carriers of reduced-function CYP2C19 allele and noncarriers separately. For multiple publications of 1 clinical trial, we will include the publication most relevant to our inclusion criteria in terms of detailed reporting of clinical vascular events.

### 2.3 Genetic eligibility criteria and outcome definition

For unambiguous determination, polymorphisms of CYP2C19 need to be designated by their rs numbers (NCBI dbSNP identifiers). We will consider reports on reduced-function variants CYP2C19∗2 (rs4244285) or CYP2C19∗3 (rs4986893), and the gain of function variants CYP2C19∗17 (rs12248560). In each study, patients taking clopidogrel are classified as carriers of reduced-function CYP2C19 genotype (heterozygotes, homozygotes, or compound heterozygotes) or noncarriers (wild-type). Of the reduced-function alleles, CYP2C19∗2 is the most frequent variant which accounts for more than 95% of the reduced-function allele carrier status in white and black African populations and for more than 75% in Asian populations.^[14,15]^ Thus, studies will be eligible if they are genotyped at least the CYP2C19∗2.

The primary outcome is the composite clinical vascular events of stroke, myocardial infarction, or vascular death, and the secondary outcome is the recurrent stroke including ischemic and hemorrhagic stroke. Ischemic stroke is defined as an episode of neurological dysfunction caused by focal cerebral, spinal, or retinal infarction. Hemorrhagic stroke is defined as rapidly developing clinical signs of neurological dysfunction and/or headache because of bleeding into the subarachnoid, brain parenchyma or ventricular system that is not caused by trauma.^[16]^ Myocardial infarction is defined according to the guidelines set forth by the third universal definition.^[17]^ Vascular death indicates any vascular-cause mortality. We will exclude studies reporting only all-cause mortality attributable to the high likelihood bias by events without an underlying vascular cause.

### 2.4 Data extraction

The data extracted from each study will include study identifiers (the name of the first author, year of publication, ethnicity); study design (case–control study, prospective cohort study, or RCT study), characteristics of study individuals (number of patients, age, sex); cardiovascular risk (hypertension, diabetes mellitus); characteristics of drug intervention (clopidogrel Loading Dose, maintenance Dose, co-medication with aspirin, duration of follow-up); CYP2C19 alleles genotype (∗2, ∗3, and ∗17); outcome measures (type and number of events in both carriers of reduced-function CYP2C19 allele and noncarriers).

### 2.5 Quality evaluation

To assess the methodological quality of randomized trials, we will adopt the guideline of the Cochrane collaboration Risk of Bias Tool.^[18]^ We will evaluate the quality of RCTs focusing on selection bias (random sequence generation, allocation concealment), information bias (masking of participants and outcome adjudicators), and bias in the analysis (incomplete outcome data and selective reporting).^[18]^ The overall risk of bias is determined as low (all analyzed items were appropriate, or at least 5 items were appropriate and the remaining 2 unclear), unclear (>2 items were not reported), and high (≥1 quality dimension suggested possible bias).^[18]^ The methodological quality of case–control study and cohort study will be evaluated using the Newcastle–Ottawa scale (NOS) criteria.^[19]^ The NOS criteria is based on 3 categories: subject selection (representativeness of the exposed cohort, selection of the nonexposed cohort, ascertainment of exposure and demonstration that the outcome of interest was not present at the start of the study); comparability of subject (comparability of cohort on the basis of the design or analysis); clinical outcome (assessment of outcome, follow-up long enough for outcome to occur, adequacy of follow-up of the cohort).^[19]^ NOS scores range from 0 to 9 with scores ≥7 indicating good quality, and poor to moderate quality when the score was < 7.^[19]^ For each study, we will also assess how the population is selected, the duration and route of medication administration, and the funding source.

### 2.6 Assessment of bias

We use the criteria described in the Cochrane Handbook of Systematic Reviews to assess for trial-level risk of bias in included studies.^[12]^ A graph of the risk of bias and a summary will be generated. Funnel plots will be generated to assess for publication bias.^[13]^

## 3 Data analysis

Statistical analyses will be performed using the STATA statistical software (version 13.0, Stata Corporation, College Station, TX). Individual studies and meta-analysis estimates will be derived and presented in forest plots. Results will be reported as odds ratios (ORs) with their 95% CIs. Heterogeneity, defined as variation beyond chance, will be evaluated through the *I*^*2*^ test that measures the percentage of total variation between studies.^[18]^ For each meta-analysis, the fixed-effects analysis will be performed according to the Mantel–Haenszel method, whereas, when *I*^*2*^ was >50%, high heterogeneity is assumed and the random-effects model will be performed according to the DerSimonian and Laird method. *P < *.05 indicate a statistically significant difference. Subgroup analyses will be performed by study of design (case–control study, cohort study, and RCTs), ethnicity (Caucasians, Asian, Black, or Hispanic), combination usage of aspirin or not, sample size, and duration of follow up. In addition, we will conduct sensitivity analyses by sequential elimination of each trial from the pool and to determine whether these changes in analysis will affect the conclusions.

## 4 Discussion

Unlike coronary artery disease, ischemic stroke is a more heterogeneous disease with various etiologies including large artery atherosclerosis, small vessel occlusion, and fragile intracranial vasculature, which is more prone to weak balance between thrombus and hemorrhage.^[16]^ In terms of long-term dual antiplatelet therapy (DAPT) versus aspirin alone, in the most recent SPS3 (the Secondary Prevention of Small Subcortical Strokes) trial (n = 3020), there is no significant reduction in recurrent stroke associated with DAPT (2.0 vs 2.4%, *P = *.12), but is associated with an increased risk of major hemorrhages (2.1 vs 1.1%, *P* < .001) and also all deaths (2.1 vs 1.4%, *P = *.004).^[20]^ Regarding the short-term DAPT versus mono-therapy, in the CHANCE trial (n = 5170), patients with minor IS or high-risk TIA who is treated with DAPT have a lower rate of the primary endpoint occurrence of ischemic or hemorrhagic stroke (8.2 vs 11.7%, *P* < .001) compared to aspirin monotherapy, whereas the rates of hemorrhagic stroke and severe bleeding (0.3 vs 0.3%, *P = *.73) does not differ between the 2 groups.^[21]^ To date, there is no evidence from randomized trials for the utility of novel P2Y12 receptor inhibitors, and clopidogrel is still the first-line treatment option for secondary stroke prevention. Clopidogrel monotherapy may be reasonable strategy for secondary prevention in stroke patients, and short-term DAPT with aspirin and clopidogrel may be more benefit in early minor IS or TIA patients whereas increased bleeding risk during long-term therapy may outweigh the ischemic benefits. In the field of coronary heart disease, CYP2C19 genotyping has been determined to become straightforwardly diagnostic approach for optimal clopidogrel treatment. Yet, there is no consensus as to whether the diminished pharmacologic response leads to increased clinical adverse events in patients with IS or TIA. The present study will focus this issue by pooling current evidence, and provide both clinical decision-making and future research. Several possible limitations of the study may be taken into account. Firstly, the studies contributing to this meta-analysis may be clinical outcomes studies in which platelet function testing is not performed. As such, we will not assess the platelet reactivity based on CYP2C19 genotype. Secondly, the majority of study population may be co-treatment with aspirin, and aspirin resistence is also the well-known reason to influence the patients’ outcome. Therefore, it is possible that more aspirin-resistant patients were included in the CYP2C19 reduced-function carriers which may impact the results. Thirdly, most of the studies included in the meta-analysis may provide information only on CYP2C19∗2. Likewise, there are other genes, not included in the present analysis, may influence the response to clopidogrel. Fourthly, our analyses will not consider co-administration with CYP2C19 inhibitors, especially proton pump inhibitors. Fifthly, we will not assess the bleeding risk of CYP2C19 reduced-function allele because of the low incidence of bleeding events in included studies. Finally, the majority of study population may be Asian, and the results may apply to other setting cautiously.

## Author contributions

ZCG and WYL conceived the idea and design for this systematic review. JCY, MC, and MMP developed the methodology for the systematic review protocol. The contents of this manuscript were drafted by ZCG with input from all members of the authorship team. The manuscript was reviewed by JCY, MC, MMP, and WYL for important intellectual content. All authors read and approved the final manuscript.

**Conceptualization:** JCY, MC, MMP, ZG, WYL.
